# Determining the Relationship Between People’s Explicit and Implicit Preferences for Gender-Inclusive Sexual and Reproductive Health Content: Randomized Controlled Trial

**DOI:** 10.2196/85868

**Published:** 2026-06-22

**Authors:** Elizabeth R Boskey, Jessica D Kant, Ariel K Berman, Frances W Grimstad

**Affiliations:** 1 Division of Gynecology, Department of Surgery, Boston Children's Hospital, 300 Longwood Ave, Boston, MA, 02115, United States, 1 617-355-7648, 1 7187011244; 2 Department of Surgery, Medical School, Harvard University, Boston, MA, United States; 3 Department of Epidemiology, T.H. Chan School of Public Health, Harvard University, Boston, MA, United States; 4 Department of Psychiatry and Behavioral Sciences, Boston Children's Hospital, Boston, MA, United States; 5 Department of Psychiatry, Medical School, Harvard University, Boston, MA, United States; 6 Department of Obstetrics, Gynecology, and Reproductive Biology, Medical School, Harvard University, Boston, MA, United States

**Keywords:** inclusive language, sexual and reproductive health, health education, randomized controlled trial, sex factors, gender identity, sex education

## Abstract

**Background:**

Inclusive health education content has been shown to increase acceptability and accessibility for lesbian, gay, bisexual, transgender, queer, intersex, and asexual, as well as other sexual and gender minority (LGBTQ+) individuals. However, there has been some backlash among general audiences, with claims that such inclusive content is “woke” or otherwise problematic.

**Objective:**

The goal of this study was to test whether individuals across the political spectrum notice when sexual and reproductive health content is written with inclusive language in order to demonstrate the acceptability of inclusive content to a broader audience.

**Methods:**

This study included 454 adults assigned female at birth from the United States, one-third of whom identified as LGBTQ+, reviewed 2 sets of reproductive health educational handouts designed for adolescents, with 1 gender-inclusive and 1 gender-specific version in each set, randomized in order. Individuals were asked to rate each document and state a preference within each pair (implicit preference). They were then debriefed on the study’s purpose and asked if they had an explicit preference for gender-specific or gender-inclusive content.

**Results:**

Preferences for explicit content tended toward gender-specific content: always gender-specific (n=184, 40.5%), sometimes gender-specific (n=59, 13%), no preference (n=131, 28.8%), sometimes gender-inclusive (n=39, 8.6%), and always gender-inclusive (n=41, 9%). However, most people (n=273, 59%) did not notice differences between the first pair of documents they viewed or rate them differently (mean difference −0.19, SD 2.17, range −10 to 12). Furthermore, the majority of individuals who had a stated preference for gender-specific health education documents did *not* choose the gender-specific document as their preferred version for either the first (n=45, 24%) or second pair of documents (n=69, 38%). Individuals who preferred content to always be gender-inclusive were significantly more likely to choose the concordant version of their document (n=20, 49% for the first pair; n=24, 58% for the second pair). A total of 58% (n=262) of the participants stated they did not notice the study design until the debrief.

**Conclusions:**

Most participants did not notice when sexual and reproductive health educational content had been made gender-inclusive—even when they had an explicit preference for gender-specific content. This suggests that when inclusive language is not directly called to readers’ attention, inclusive sexual and reproductive health content is broadly acceptable to individuals across a range of political beliefs. The use of inclusive language may therefore be a means of increasing the accessibility and applicability of educational materials to diverse recipients, including LGBTQ+ individuals.

## Introduction

In the early 2000s, as acceptance for lesbian, gay, bisexual, transgender, queer, intersex, and asexual, as well as other sexual and gender minority (LGBTQ+) individuals was growing across the population of the United States, there was increasing awareness of the need to create sexual and reproductive health education content that would be more inclusive of sexuality and gender [1-3]. One way to accomplish this is by enhancing patient educational materials through reducing or removing assumptions about the gender identity of individuals being discussed, and for sexual health materials, also removing assumptions about the genders of discussed sexual partners, that is, making the content inclusive. This approach has also been described as degendering health content. Over time, language options were developed for these materials to be more inclusive of a broad range of gender identities and sexual orientations (eg, pregnant person, birthing parent, lactating parent, sperm-producing partner, person with a uterus, ovaries, vagina, penis, etc). This often involved focusing on anatomy or experience rather than identity, as those aspects of life tended to be more relevant to the health question or behavior of interest [4]. Inclusive language makes sexual and reproductive health content more relevant to and actionable for lesbian, gay, bisexual, and transgender individuals rather than reinforcing notions that these aspects of a healthy life are only accessible and attainable to those who are cisgender and heterosexual [5-7].

The development of inclusive content has frequently been accompanied by opposition, often politically and/or religiously driven [8-10]. This opposition is often based on inaccurate beliefs that all individuals who have the same anatomy (eg, uterus) will have the same gender (eg, woman). This is problematic not just because up to 2% of the population identify as transgender [11], but because other women may have been born without a uterus or had it removed. In recent years, there has been an exponential growth in attacks on gender and sexual diversity across media and government [12-14]. This has peaked under the second Trump administration, with numerous executive orders and policy initiatives designed to erase transgender and other gender diverse individuals from public life [15-17]. There is also sometimes a perception that it is necessary to safeguard women-centric health care with women-centric language [18,19]. This is particularly true in obstetrics and gynecology—even though not all aspects of women’s health are nested within obstetrics and gynecology and not all patients who see obstetricians and gynecologists are women

For many individuals whose sexuality, anatomy, and/or gender differ from expected binary norms, including those who may have been born with variations in their sexual and reproductive anatomy (eg, without a uterus) or have undergone surgeries to alter it (eg, a hysterectomy), health education content that assumes gender based on anatomy, or anatomy based on gender, may not come across as accurate, relevant, or useful, and thus fail to meet their needs. As such, it may be helpful to develop sexual and reproductive health content that is anatomy and behavior-specific and not reliant upon assumptions about gender or sexuality [20]. However, that usefulness is dependent on inclusive medical content not being off-putting, offensive, or considered unrelatable, to heterosexual individuals whose anatomy and gender do reflect binary norms.

In response to community calls for greater uptake of inclusive content, some individuals have expressed strong feelings and beliefs about the dangers or offensiveness of such content [21]. However, to date, no studies have examined whether those opposed to inclusive language notice when such language is present but not specifically brought to their attention. This randomized controlled trial was therefore designed to test the hypothesis that most individuals do not notice when sexual and reproductive content has been changed to use inclusive language, with the exception of those for whom inclusive language is more personally relevant. It was also designed to test whether political identity and/or LGBTQ+ identity were associated with explicit preferences for gender-specific versus inclusive content and/or the likelihood of noticing and preferring such content.

## Methods

### Study Population

The Prolific survey platform [22] was used to recruit individuals assigned female at birth who lived in the United States for a study in which they were informed that they would be shown 2 different versions of online health content and asked which they preferred. In order to ensure respondents are genuine, potential participants must provide a photo taken with a state or federal ID when registering for the platform. The site validates IP addresses to prevent duplication, uses machine learning to screen for potential automation, and allows studies to withhold payment for suspicious responses, including those completed substantially faster than the average time for completion.

The Prolific quota function was used to recruit a population that was approximately half LGBTQ+-identified and equally divided among conservative, moderate, and liberal political orientations based on the site’s built-in demographic questionnaires. Individuals were also asked to self-identify within the survey, and those identifications were used in the analysis. Because of this, the actual sample demographics were not identical to the planned quota sample, reflecting the fact that individuals may have completed initial screeners for the Prolific platform up to several years prior to any given survey participation and some of their answers may have changed.

### Study Design

Individuals were asked to review 2 versions of 2 sets of patient-educational materials that were originally designed for adolescents assigned female at birth—the first on polycystic ovarian syndrome and the second on birth control. Within each set, 1 document was made inclusive, while the other used gender-specific language. Two study team members reviewed language differences in the documents to ensure consensus on language choice. The documents were otherwise identical, except that one (redundant) figure in the inclusive birth control document was removed due to the use of images of pregnant women in the figure (changes are listed in Table 1). Individuals were randomized using an allocation table generated in Excel, so that half received the inclusive document first in each set, while the other half received the explicitly gendered material first.

**Table 1.** Text changes made from gender-specific to gender-inclusive document versions.

Context for change (if needed)	Gender-specific version	Gender-inclusive version
PCOS^a^ (~1550 words)		
PCOS is a common problem among…	girls and young women	people who have ovaries
Almost 1 out of 10…	women have PCOS	people who have ovaries have PCOS
Section Heading:	Female reproductive anatomy	Reproductive anatomy of someone with a uterus and ovaries
The ovaries make estrogen and progesterone…	(the female sex hormones)	[deleted]
3x in document	women and girls with PCOS	people with PCOS
10x in document	women	people
… but if you have PCOS, your ovaries make a little bit more testosterone than they are supposed to	All women make testosterone	Everyone makes testosterone
--	teenage girls	adolescents
Birth control (~1400 words)		
--	female-controlled contraception	contraception
These are similar to the estrogen and progesterone normally made by []	women’s ovaries	the ovaries
--	female hormones	estrogen and progestin
the male’s sperm	sperm
some [] can’t take or tolerate estrogen	girls and women	people
8x in document	women	people
--	condoms	condoms or other barrier methods
Perfect use can be difficult for…	teen girls and adult women	teens and adults
Very occasionally, your breasts may become tender and/or get larger, but usually your breasts will stay the same. []	Breast tenderness	This tenderness
Can any [] take birth control pills?	woman	person born with a uterus and ovaries
Almost all [] can take birth control pills	women	people born with a uterus and ovaries
Table describing pregnancy rates associated with birth control pill use	Has images of pregnant silhouettes as illustrations of the number of pregnancies, alongside text	Text only

a PCOS: polycystic ovary syndrome, name since changed to PMOS (polyendocrine metabolic ovarian syndrome).

After reading each document, individuals were asked to rate the content on a 5-point Likert scale (−2 to +2) across the following domains: easy to understand, reliable, biased, meant for people like me, and relevant to people like me. These scores were then summed to provide an overall score for each document, with the bias question reverse scored. Individuals were also allowed to comment on each rating. After reviewing each pair of documents, individuals were asked if they had a version preference and to explain their reasoning. A separate comparison score was generated by subtracting individuals’ gender-inclusive score from the gender-specific score, so that higher comparison scores were associated with stronger implicit preferences for the gender-specific version.

Finally, after reviewing both sets of informational material, individuals completed a demographic survey that concluded with a question about whether sex education should be required as part of public K-12 education. They were then presented with a debrief that explained the purpose of the study, which included asking whether they had noticed that they were being presented with gender-inclusive material and if they preferred material to be gender-specific or gender-inclusive (explicit preference). Individuals were also given the opportunity to provide their closing thoughts. The questions are provided as a supplementary document. Individuals were not able to return to previous sections of the study or revise their answers to questions. All materials were accessed via Research Electronic Data Capture [23,24].

### Ethical Considerations

This study was approved by the Boston Children’s Hospital IRB (IRB-P00047950).

### Analysis

#### Power Calculation

The study was powered based on the use of a parallel 2-group design to test whether group A (gender-inclusive first) mean (μ1) was equivalent to group B (gender-specific first) mean (μ2), with difference equivalence bounds of −1 and 1 (H0: *δ* ≤−1 or *δ* ≥1 vs H1: −1<*δ*<1, *δ* = μ1–μ2), with an overall type I error rate (α) of .05. The common SD was assumed to be the fixed value of 2, with the actual SD for the 2 comparison scales ranging from 2.2 to 2.3. To detect a mean difference (*δ* = μ1–μ2 = μT–μR) of 0 with sample sizes of 225 for group 1 (treatment) and 225 for group 2 (reference), the power was .9997. With the actual sample breakdown of 176 in the LGBT+ group and 278 in the cisgender-heterosexual group, the power was calculated as .9996 to determine equivalence between those groups. The power was computed using PASS 2023 (version 23.0.7).

#### Quantitative Analysis

Quantitative analyses were performed in Stata 18.0 (StataCorp LLC). Demographic variables are presented as numbers and percentages. Differences across categories of explicit, stated preferences for gender-inclusive information were calculated using the chi-square test.

For each document, pair preferences for those with varying explicit preferences were categorized as concordant (ie, states they prefer gender-specific information, chose gender-specific document as preferred) or discordant or do not notice (ie, either stated there was no difference between the documents or chose the type of document they said they did not prefer). Differences across explicit preference categories were calculated using the chi-square test as well as for when individuals stated they noticed that 1 document in each pair was gender-inclusive. Differences in calculated preference scores for each pair of documents were analyzed using ANOVA.

#### Qualitative Analysis

Open-ended answers were analyzed in the Dedoose cloud app for managing, analyzing, and presenting qualitative and mixed method research data (Version 9.2.22: Sociocultural Research Consultants, LLC). Excerpts were excluded from analysis if they did not provide additional information (ie, were a repeat of the answer from a numeric field, or contained a one-word answer such as “no,” “yes,” or “useful”). They were also excluded if they stated a fact unrelated to the question of interest (eg, “This content is not relevant to me because I do not use birth control pills”). Due to the brevity of responses, inductive content analysis was used to identify commonalities between comments, as well as whether this varied by politics and/or stated (explicit) preference for gender-inclusive information [25,26]. Codes were first generated inductively and then grouped by frequency to provide an overview of types of responses. Summative or frequency information is additionally provided to demonstrate between-group differences.

## Results

### Overview

**Figure 1.**
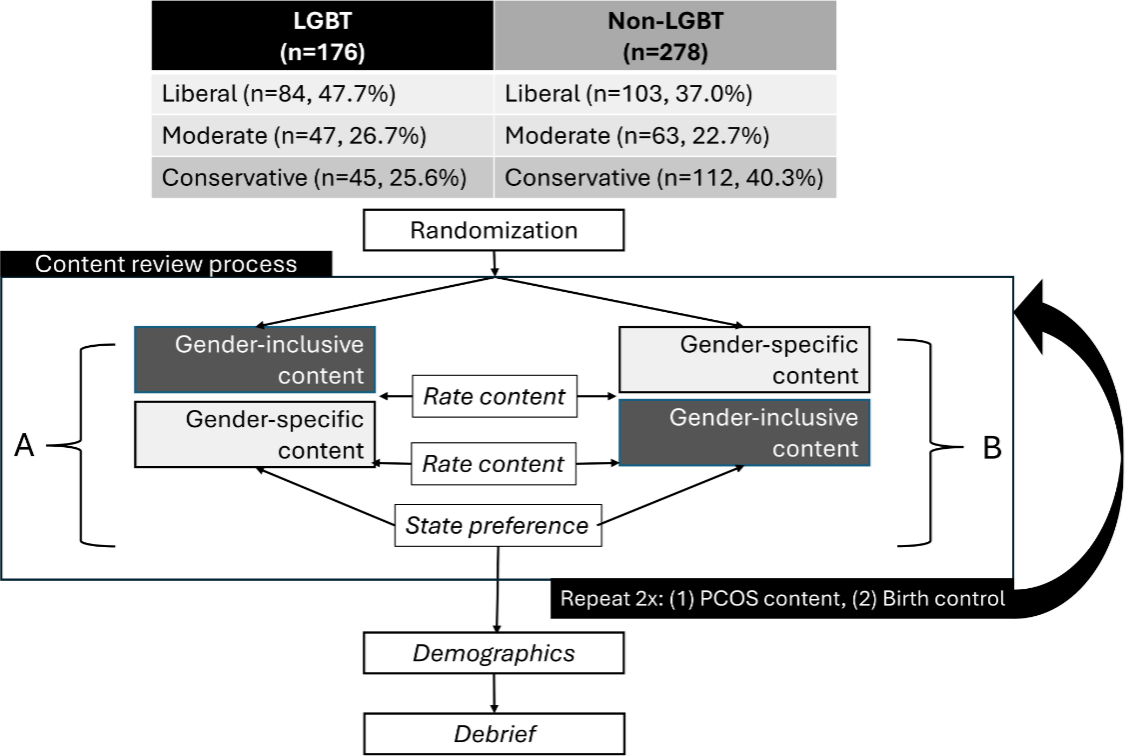
Study flow design. Each assessment is listed in italics. Simultaneously attempting to make quotas on both LGBT identity (1:1) and political identity (1:1:1) led to slightly unbalanced samples, although with sufficient diversity for analyses. LGBT: lesbian, gay, bisexual, transgender; PCOS: polycystic ovary syndrome.

In total, 494 individuals participated in the study, 227 in the version that received the gender-inclusive documents first and 227 in the version that received the gender-specific documents first. There were 40 people who started the study but did not complete it, resulting in a response or completion rate of 92% (n=454). The study flow is outlined in Figures1  2.

**Figure 2.**
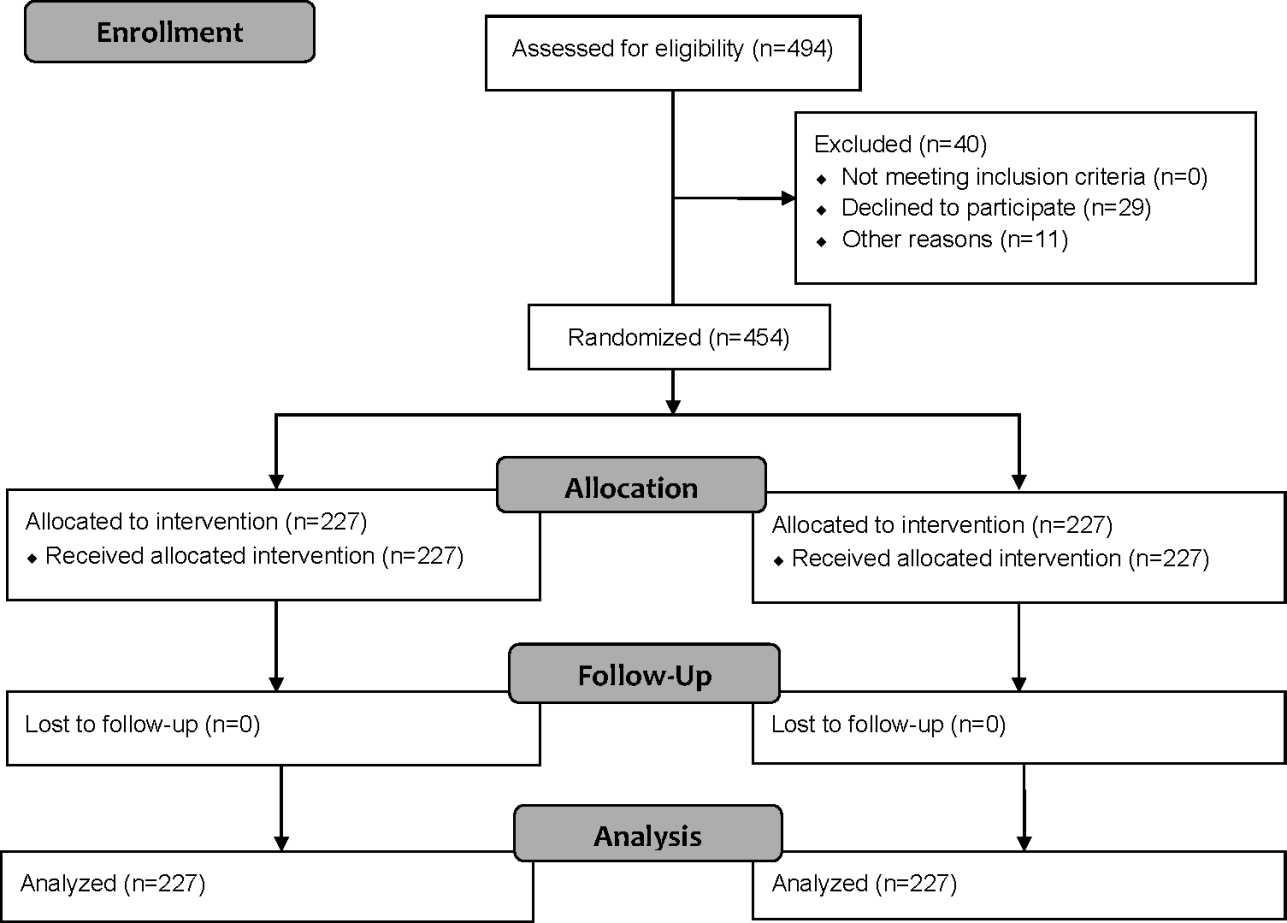
CONSORT (Consolidated Standards of Reporting Trials) 2010 diagram. Checklist is available as Checklist 1.

Participants were 41% (n=187) liberal, 24% (n=110) moderate, and 35% (n=157) conservative; 39% (n=176) were LGBTQ+, with 18% (n=32) identifying as transgender and/or nonbinary. These numbers do not match the intended quotas because individuals’ self-identification in terms of politics and/or sexual orientation and gender identity may have changed since they filled out the screeners on the survey platform. (Table 2; Figure 1)

**Table 2.** Demographics of study participants, by explicit preference for gender-inclusive versus gender-specific information.^a^

Demographics	Always gender-specific (n=184), n (%)	Sometimes gender-specific (n=59), n (%)	No preference (n=131), n (%)	Sometimes gender-inclusive (n=39), n (%)	Always gender-inclusive (n=41), n (%)	Chi-square (*df*); *P* value
How old are you? (y)						28.9 (16); .02^b^
18‐24	29 (15.8)	12 (20.3)	11 (8.4)	7 (17.9)	6 (14.6)	
25‐34	48 (26.1)	16 (27.1)	49 (37.4)	16 (41.0)	15 (36.6)	
35‐44	43 (23.4)	15 (25.4)	37 (28.2)	8 (20.5)	13 (31.7)	
45‐54	32 (17.4)	14 (23.7)	25 (19.1)	4 (10.3)	3 (7.3)	
55‐64	32 (17.4)	2 (3.4)	9 (6.9)	4 (10.3)	4 (9.8)	
Which of the following best describes your racial/ethnic background?						33.7 (28); .21
White	112 (60.9)	40 (67.8)	96 (73.3)	23 (59.0)	35 (85.4)	
Black	46 (25.0)	13 (22.0)	16 (12.2)	13 (33.3)	4 (9.8)	
Hispanic	7 (3.8)	2 (3.4)	10 (7.6)	0 (0)	1 (2.4)	
Asian or Pacific Islander	6 (3.3)	2 (3.4)	1 (0.8)	2 (5.1)	0 (0)	
Native American	2 (1.1)	0 (0)	1 (0.8)	0 (0)	1 (2.4)	
Multiracial	9 (4.9)	2 (3.4)	7 (5.3)	1 (2.6)	0 (0)	
Something else	1 (0.5)	0 (0)	0 (0)	0 (0)	0 (0)	
Prefer not to answer	1 (0.5)	0 (0)	0 (0)	0 (0)	0 (0)	
Were you born in the United States?						10.2 (8); .25
Yes, and so were my parents	151 (82.1)	49 (83.1)	115 (87.8)	31 (79.5)	39 (95.1)	
Yes, but my parents were not	21 (11.4)	4 (6.8)	10 (7.6)	3 (7.7)	1 (2.4)	
No	12 (6.5)	6 (10.2)	6 (4.6)	5 (12.8)	1 (2.4)	
Highest level of education achieved						47.1 (28); .01^b^
Less than high school	0 (0)	0 (0)	2 (1.5)	0 (0)	2 (4.9)	
High school diploma or General Educational Development (GED) test	17 (9.2)	6 (10.2)	29 (22.1)	4 (10.3)	2 (4.9)	
Some college	29 (15.8)	10 (16.9)	24 (18.3)	5 (12.8)	10 (24.4)	
Associate’s degree	22 (12.0)	7 (11.9)	15 (11.5)	2 (5.1)	9 (22.0)	
Bachelor’s degree	79 (42.9)	20 (33.9)	39 (29.8)	17 (43.6)	11 (26.8)	
Master’s degree	29 (15.8)	14 (23.7)	19 (14.5)	11 (28.2)	5 (12.2)	
Professional degree	2 (1.1)	0 (0.0)	2 (1.5)	0 (0)	1 (2.4)	
Doctorate degree	6 (3.3)	2 (3.4)	1 (0.8)	0 (0)	1 (2.4)	
Which of the following best describes your political orientation?						154.3 (16); <.001^b^
Very Liberal	11 (6.0)	10 (16.9)	37 (28.2)	17 (43.6)	27 (65.9)	
Somewhat Liberal	25 (13.6)	16 (27.1)	28 (21.4)	7 (17.9)	9 (22.0)	
Moderate	37 (20.1)	19 (32.2)	44 (33.6)	6 (15.4)	4 (9.8)	
Somewhat Conservative	56 (30.4)	9 (15.3)	17 (13.0)	2 (5.1)	1 (2.4)	
Very Conservative	55 (29.9)	5 (8.5)	5 (3.8)	7 (17.9)	0 (0.0)	
Which of the following comes closest to how you think about the place of religion in your life? [Religion is…]						96.8 (16); <.001^b^
The most important thing	52 (28.3)	5 (8.5)	8 (6.1)	2 (5.1)	1 (2.4)	
One among many important things	78 (42.4)	21 (35.6)	32 (24.4)	14 (35.9)	8 (19.5)	
Not as important as other things	21 (11.4)	9 (15.3)	19 (14.5)	6 (15.4)	3 (7.3)	
Not important	31 (16.8)	24 (40.7)	68 (51.9)	16 (41.0)	25 (61.0)	
Prefer not to answer	2 (1.1)	0 (0)	4 (3.1)	1 (2.6)	4 (9.8)	
Do you routinely visit a gynecologist and/or get gynecological/reproductive health care?						16.3 (12); .18
Yes, at least once every two years	116 (63.0)	34 (57.6)	69 (52.7)	25 (64.1)	21 (51.2)	
Yes, at least once every five years	29 (15.8)	6 (10.2)	22 (16.8)	4 (10.3)	6 (14.6)	
No, but I have in the past	28 (15.2)	16 (27.1)	35 (26.7)	8 (20.5)	8 (19.5)	
No, and I prefer to not get this type of care	11 (6.0)	3 (5.1)	5 (3.8)	2 (5.1)	6 (14.6)	
Do you consider yourself to be LGBTQ+^c^ (ie, a sexual or gender minority)?						27.7 (8); <.001^b^
No	132 (71.7)	31 (52.5)	70 (53.4)	23 (59.0)	16 (39.0)	
Yes	51 (27.7)	27 (45.8)	59 (45.0)	14 (35.9)	25 (61.0)	
I don’t know	1 (0.5)	1 (1.7)	2 (1.5)	2 (5.1)	0 (0)	
Do you identify as transgender and/or nonbinary?						28.3 (8); <.001^b^
No	44 (86.3)	23 (85.2)	51 (87.9)	8 (57.1)	12 (48.0)	
Yes	6 (11.8)	2 (7.4)	7 (12.1)	5 (35.7)	12 (48.0)	
I don’t know	1 (2.0)	2 (7.4)	0 (0)	1 (7.1)	1 (4.0)	
Do you think that sex education should be required as part of public K-12 education?						62.4 (16); <.001^b^
Yes, and it should be comprehensive	100 (54.6)	45 (76.3)	94 (72.3)	31 (79.5)	41 (100)	
Yes, and it should emphasize abstinence until marriage	19 (10.4)	2 (3.4)	10 (7.7)	2 (5.1)	0 (0)	
Maybe, and if offered it should be comprehensive	21 (11.5)	8 (13.6)	21 (16.2)	3 (7.7)	0 (0)	
Maybe, and if offered, it should emphasize abstinence until marriage	25 (13.7)	2 (3.4)	2 (1.5)	3 (7.7)	0 (0)	
Sex education should not be allowed in public schools	18 (9.8)	2 (3.4)	3 (2.3)	0 (0)	0 (0)	

a The column headings represent the options for the following item: “When thinking about the health information that you read online or get from a health care provider, do you like it to be:”. Differences across these categories were calculated using the chi-square test.

b Statistically significant at *P<*.05.

c LGBTQ+: lesbian, gay, bisexual, transgender, queer, intersex, and asexual, as well as other sexual and gender minority.

### Participants Stated (Explicit) Preferences Around Gender-Inclusive Language

After individuals were debriefed, and the purpose of the study was explained, they were asked, “When thinking about the health information that you read online or get from a health care provider, would you like it to” and then given the choices of “always be gender-specific (eg, women’s health, pregnant women, mother, etc)” (n=184, 40.5%), “sometimes be gender-specific” (n=59, 13%), “no preference either way” (n=131, 28.8%), “sometimes be degendered (eg, reproductive and sexual health, pregnant people, parent, etc)” (n=39, 8.6%), or “always be degendered” (n=41, 9%). We refer to this as their stated or explicit preference for the remainder of the analysis.

Individuals who stated they preferred inclusive language tended to be younger (*χ*^2^_16_=28.9; *P=*.02) and more liberal (*χ*^21^_16_=154.3; *P<*.001) and also considered religion less important (*χ*^2^_16_=96.8; *P<*.001) than those who preferred gender-specific language. They were also more likely to be LGBT (*χ*^2^_8_=27.7; *P<*.001) and specifically transgender or nonbinary (*χ*^2^_8_=28.3; *P<*.001). Most strikingly, the differences in preference were strongly associated with differences in perspectives on sex education in public schools, which was used as a proxy for people’s overall beliefs on reproductive health education (*χ*^2^_16_=62.4; *P<*.001). Every person who preferred inclusive language supported comprehensive sex education in public schools, while no one who thought sex education should not be allowed in public schools had an explicit preference for inclusive language.

### People’s Awareness of Inclusive Language

Of the people who saw the inclusive versions first, 56.9% (n=132) stated they did not notice a difference between the documents in the first pair, and 41.4% (n=94) stated they did not notice a difference between the documents in the second pair. These numbers were 61.6% (n=141) and 42.2% (n=96) for those who saw the gender-specific versions first. After the debrief, when asked explicitly about when they noticed that some of the documents were inclusive, 57.7% (n=262) stated that they did not notice until the debrief, 21.1% (n=96) stated that they noticed after the first pair, and 21.1% (n=96) stated that they noticed after the second pair. This did not differ by version (*χ*^2^_2_=0.6; *P=*.75). Individuals with the strongest preferences (ie, always) tended to notice sooner than those with weaker (ie, sometimes) or no preference (*χ*^2^_8_=56.3; *P<*.001; Table 3).

**Table 3.** Relationship between stated (explicit) preference for gender-specific versus gender-inclusive content at the time of debrief and measured content preferences.^a^

Stated preference for pair	Always be gender-specific (n=184)	Sometimes be gender-specific (n=59)	No preference (n=131)	Sometimes be gender-inclusive (n=39)	Always be gender-inclusive (n=41)	Chi-square or ANOVA F (*df*); *P* value
PCOS^b^, n (%)						11.2 (3)^c^; .01
Concordant preference^d^	45 (24.5)	13 (22.0)	—^e^	10 (25.6)	20 (48.8)	
Do not notice or discordant preference	139 (75.5)	46 (78.0)	—	29 (74.4)	21 (51.2)	
Birth control, n (%)						9.8 (3)^c^; .02
Concordant preference	69 (37.5)	25 (42.4)	—	10 (25.6)	24 (58.5)	
Do not notice or discordant preference	115 (62.5)	34 (57.6)	—	29 (74.4)	17 (41.5)	
When did you notice that one of each set of the content was gender-inclusive? n (%)						56.3 (8)^c^; <.001
After first	53 (28.8)	9 (15.3)	8 (6.1)	10 (25.6)	16 (39.0)	
After second	36 (19.6)	13 (22.0)	20 (15.3)	16 (41.0)	11 (26.8)	
At debrief	95 (51.6)	37 (62.7)	103 (78.6)	13 (33.3)	14 (34.1)	
Preference scores (higher=stronger preference for gender-specific), mean (SD)						
Content 1 (PCOS)	0.6 (2.4)	0.3 (2.4)	0.0 (1.1)	0.3 (1.7)	−1.4 (2.9)	11.5 (1)^f^; <.001
Content 2 (birth control)	0.2 (2.3)	−0.1 (2.1)	−0.0 (1.7)	0.2 (2.2)	−1.7 (3.2)	8.2 (1)^f^; <.001

a The column headings represent the options for the following item: “When thinking about the health information that you read online or get from a healthcare provider, do you like it to.”

b PCOS: polycystic ovary syndrome.

c Chi-square.

d For always or sometimes gender-specific, a concordant preference was defined as preferring the gender-specific document. For always or sometimes gender-inclusive, a concordant preference was defined as preferring the gender-inclusive document.

e Not applicable, individuals who did not have an explicit preference were excluded from this portion of the analysis.

f ANOVA F.

### People’s Version Preferences in Relation to Their Stated (Explicit) Preferences

**Figure 3.**
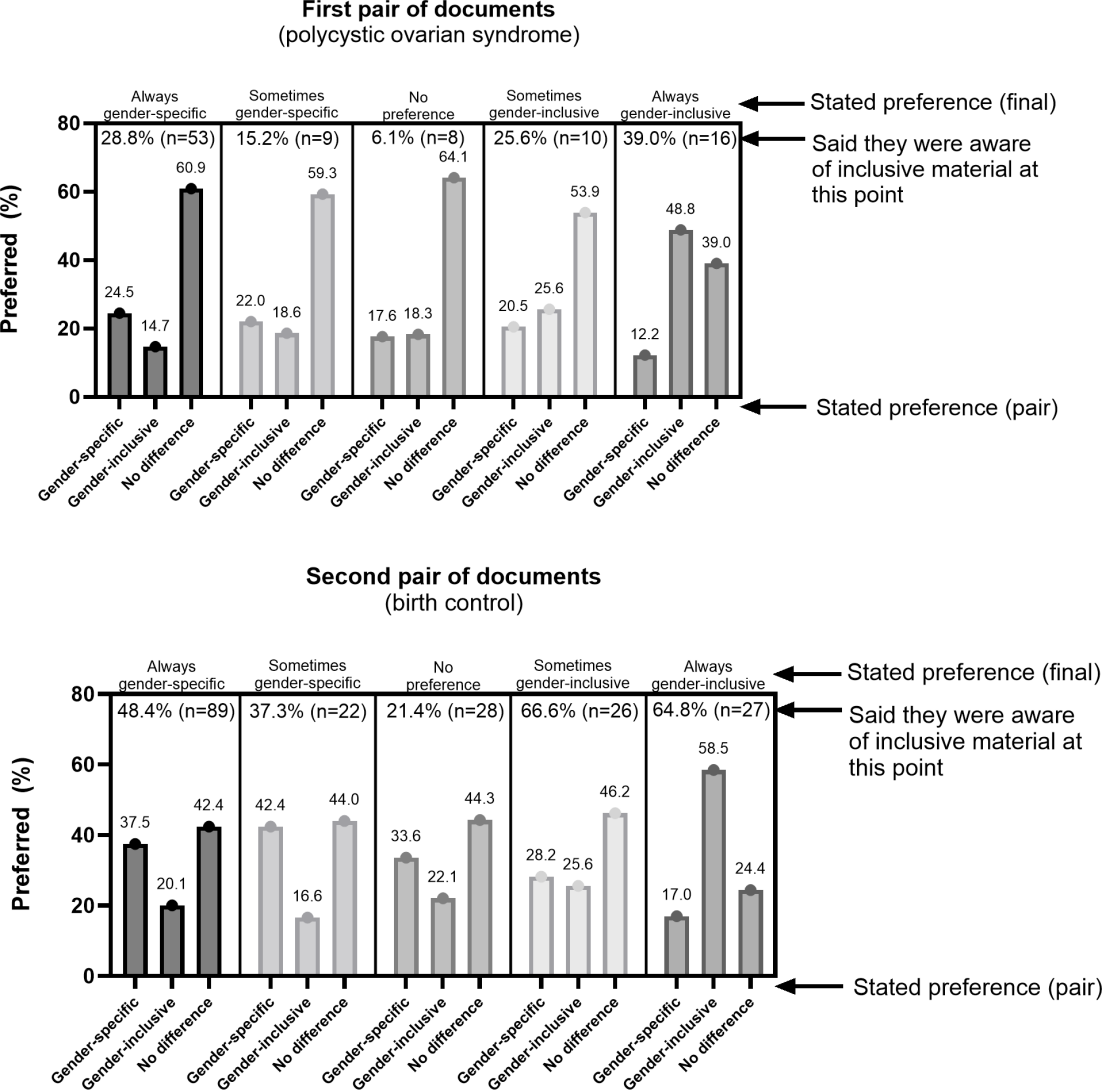
Stated preferences for each pair of documents (bars) organized by the final explicit preference stated for gender-specific versus gender-inclusive content (sections). The percentage of individuals who stated, at the end of the study, that they were aware of degendering after viewing the document pair is shown at the top of each section.

Looking just at individuals who had a stated preference for gender-specific or gender-inclusive documents (ie, excluding those who chose “no preference”), the majority of individuals who had a stated preference for gender-specific health education documents *did not choose* the gender-specific document as their preferred version for either the first or second pair of documents, although the number of individuals with a concordant preference did increase for the second document pair. The individuals who preferred content to always be inclusive were substantially more likely to choose the concordant version of their document than those from other stated preference groups (n=20, 48.8% for the first pair; n=24, 58.5% for the second pair). This pattern was also evident in the preference scores generated by questions asked after each individual document, where the only group with large differences in rating scores between the paired versions was those who wanted content to always be inclusive (Table 3, Figure 3).

### Qualitative Analysis of Open-Ended Responses

We hypothesized during study design and data collection that most participants who preferred gender-specific language would not notice inclusive language, even when they wanted the language to always be gender-specific. While this was true in the quantitative responses, analysis revealed that some people felt strongly about minor differences between the documents. This manifested qualitatively in the free-text responses, which were categorized using inductive content analysis (Multimedia Appendix 1).

When reviewing the comments on why individuals preferred their chosen versions, the category with the greatest number of comments was “perception versus reality,” dominated by those who appeared to perceive substantially more differences than were actually present between document versions (n=82), which they used to justify their preferences (ie, citing things such as different layouts, flow, and comprehensiveness). That category also included individuals commenting during the debrief, usually with surprise, that they had not noticed the difference between the pairs of documents (n=21). The next most common category of comment was those related to discourse including comments mentioning inclusiveness (n=33), bioessentialism (n=26), and the use of specific language from media tropes (n=11). Finally, there were groups of comments evaluating specific types of language (gender-inclusive, n=27; gender-specific, n=14; problems with inclusive, n=12; pros and cons of both, n=8), personal preferences for gender-specific (n=14) versus gender-inclusive (n=13) content, and comments mentioning identity or writing quality (Multimedia Appendix 2).

## Discussion

### Principal Findings

Confirming our primary hypothesis, the majority of study participants who were opposed to the use of inclusive language either did not notice a difference between gender-specific and gender-inclusive materials or preferred the materials with inclusive language. Those individuals who had a strong preference for inclusive information were significantly more likely to both notice the differences and choose the concordant document than those with other preferences. Nonetheless, it is important to acknowledge that half of that group still did not notice a difference between the materials when the first pair was presented to them.

The study results also confirmed our secondary hypothesis. Individuals had stronger explicit or stated preferences about gender-inclusive language than were represented by their actual ratings and choices of different types of content. In other words, it appeared that people had opinions about this issue even when the presence of inclusive language did not affect them in practice. This was demonstrated by the fact that what people stated as their explicit preference for gender-specific information did not actually predict the content they said they preferred, but it did predict their political views.

Our data suggest that individuals may hold beliefs about the concept of inclusion that differ from the reality of how they experience it. For some people, this may reflect their having been influenced by media and other forms of social polarization to be upset about the concept of inclusion rather than the reality of it. It is also possible that outside of the echo chamber of highly polarizing virtual communities, the importance or performance of highly gendered and explicitly anti-inclusive preferences is reduced. The act of engaging with potentially applicable health content about the *real self* may support different attention to that content than the *virtual self* who may be more engaged with anti-inclusion rhetoric [27]. That said, there were a minority of participants who did appear to be primed for identifying inclusive content, some of whom reproduced common, inaccurate comments about gender and sex in their open-ended responses.

The fact that most people did not appear to perceive a difference between gender-inclusive and gender-specific content when they had not been primed to look for it suggests that discourse in the United States about the potential harms of inclusive language, often amplified by self-described ultra-conservatives or right-wing groups, does not reflect the lived realities of most Americans. Hyper-politicization of diversity, equity, and inclusion (DEI) can lead to discussions of these concepts in the context of health care inducing stronger emotional reactions than their actual implementation would [21,28,29]. Our research suggests that, in the coming months and years, it may be important to universally adopt inclusive language as a change in nomenclature that promotes accuracy and does not have significant bearing on the way in which critical health information is perceived by the majority of patients, rather than as an explicitly DEI approach.

### Future Directions

In the future, we hope to analyze the use of inclusive language in real-world situations and in populations of younger patients and their caregivers. Our intention is to decouple the perception of inclusive language from DEI efforts and instead focus on it as a way of accurately and nonjudgmentally describing the bodies of individuals receiving care—similar to the use of accurate anatomic terminology. We also intend to test whether inclusive language is received differently when used in combination with gender-specific language versus on its own (eg, *women and other* people with ovaries vs *people with ovaries*). We hypothesize that combination language will have broader acceptability but possibly also lead to stronger negative reactions in a small minority of people at both ends of the political spectrum—who could become primed to notice and respond to the presence of inclusive language. Our next step in doing so will involve focus groups with a diverse range of people—exploring their reactions to these research results to understand their interpretation and the implications for dissemination. We would also like to use this method to examine people’s responses to changing sex and gender language in information unrelated to health—for example, information on the benefits of sports participation for people of *all sexes* versus *all genders*. We hypothesize that subject matter, salience, and valence may all play a role in an individual’s response.

### Strengths and Limitations

The strengths of this study include a robust study design and a large, diverse respondent sample. The sample also clearly engaged with the research. This could be seen in the extensive comments across open-ended fields.

The main limitation of this study is that it is impossible to rule out rendering differences that may have contributed to the perception of some users that there were substantial differences between the 2 sets of virtually identical documents. The open-ended responses both confirmed and disconfirmed this. Some participants reported that they had believed the 2 documents in each set to be largely identical. Others seemed to believe that the content was completely different, describing different formatting, different lengths, 1 version being much easier to understand, and so forth.

Because the perception of difference among some users was so substantial, care was taken to replicate the study on multiple machines, varying device types (eg, phone, computer), operating systems, and browsers. Across systems, the only identifiable differences between the materials were the minor differences detailed in Table 1.

The study was also, potentially, limited by the sociopolitical environment in which it took place. Participants completed the study in late December 2024, after the election of Donald Trump but before his inauguration. This may have contributed to experiences of polarization among participants. The study was also specifically designed to capture a politically broad range of the US population and cannot be extrapolated to populations outside the United States.

Because of the sampling choice to purposively recruit for political and LGBTQ+ diversity over other forms of diversity, the sample is not an accurate representation of the full diversity of the United States. For example, looking at the racial and ethnic makeup of the United States, the proportions of the White (67% vs 59%), Black (20% vs 13%), and multiracial (4% vs 2%) populations were higher in this sample than reported in 2022 Census data, while the Hispanic (4% vs 19%) and Asian (2% vs 6%) populations were lower, and that of American Indian or Alaskan Native population (0.8% vs 0.7%) were similar [30].

### Conclusions

Despite substantial conservative discourse about the ways in which gender-inclusive sexual and reproductive information is offensive and harmful to women, the majority of individuals assigned female at birth do not appear to notice when such content has been made inclusive—even when they state an explicit preference for gender-specific content. The exception is those individuals for whom inclusive content is explicitly relevant or preferred, who both notice and choose content that is concordant with their preferences at much higher rates than those who prefer content to be gender-specific. This suggests that, by and large, inclusive health education is broadly useful and acceptable.

## Supplementary material

10.2196/85868 Multimedia Appendix 1 Major categories and codes derived from inductive content analysis of open-ended responses provided by participants.

10.2196/85868 Multimedia Appendix 2 Content analysis of open-ended responses provided by participants.

10.2196/85868 Checklist 1 CONSORT checklist.

## Abbreviations

CONSORT: Consolidated Standards of Reporting Trials
DEI: diversity, equity, and inclusion
LGBTQ+: lesbian, gay, bisexual, transgender, queer, intersex, and asexual, as well as other sexual and gender minority
