# Supplementary material for: Determining the Relationship Between People’s Explicit and Implicit Preferences for Gender-Inclusive Sexual and Reproductive Health Content: Randomized Controlled Trial
Source: Interact J Med Res. 2026 Jun 22;15:e85868. doi: 10.2196/85868 (PMC13286073; doi:10.2196/85868)
Supplement: Multimedia Appendix 1 [file ijmr-v15-e85868-s001.docx]

**Supplementary Table 1:** Major categories and codes derived through inductive content analysis of open-ended responses provided by participants.

| **Category** | **Category definition** | **Code** | **Code definition** |
| --- | --- | --- | --- |
| Perception vs. Reality | Comments suggest discordance between written and perceived content | Sees more differences | Discusses changes/differences that are not present in the document |
|  |  | Did not notice a difference | Mentions not noticing a difference between versions |
| Discourse | Speaks to specific tropes within (primarily anti-trans) discourse | Inclusiveness | Mentions inclusiveness |
|  |  | Bioessentialism | Gender is binary and fully determined by sex assigned at birth |
|  |  | Trans/non-binary inclusiveness | Mentions specifically trans/non-binary inclusiveness |
|  |  | Media Tropes | Incorporates non-standard language or concepts appearing most often in politicized (primarily anti-trans) media |
|  |  | Emotional valence | Text indicative of strong feelings, often by capitalization and punctuation choices |
|  |  | “Scientific Accuracy” | Uses (incorrect) claims that content is not scientifically accurate if gender-inclusive language used |
| Inclusiveness evaluation | Beliefs about superiority of gendered or degendered content | Benefits of inclusive | Provides reasons for why inclusive language is good, useful, etc. |
|  |  | Benefits of gender-specific | Provides reasons for why gender-specific language is good, useful, etc. |
|  |  | Does not want inclusive | Discusses reasons why inclusive content is incorrect, inappropriate, etc. |
|  |  | Balanced pros and cons | Discusses pros and cons for both gender-specific and gender-inclusive language |
| Personal preference | Stated preference in content type for self | Wants gender-specific | Prefers gender-specific content |
|  |  | Wants inclusive | Prefers inclusive content |
| identity | Mentions an identity referent | LGBTQ+ | Identity as a sexual or gender minority person or as intersex |
|  |  | Trans | Identity as specifically transgender or non-binary |
|  |  | Woman | Identity as a woman |
| Writing quality | Stylistic or grammatical notation | Clarity/wordiness | Expresses concerns about clarity of content related to grammar, wordiness, length, or similar issues rather than the specific material |
