# Supplementary material for: Determining the Relationship Between People’s Explicit and Implicit Preferences for Gender-Inclusive Sexual and Reproductive Health Content: Randomized Controlled Trial
Source: Interact J Med Res. 2026 Jun 22;15:e85868. doi: 10.2196/85868 (PMC13286073; doi:10.2196/85868)
Supplement: Multimedia Appendix 2 [file ijmr-v15-e85868-s002.docx]

**Supplementary Table 2:** Content analysis of open-ended responses provided by participants. Codes are listed from most common to least common, by category, with representative excerpts. Codes that occurred fewer than five times are excluded from the table. Where excerpts are trimmed, it is noted with ellipses. Clarifications are in italics. Spelling and capitalization are as typed by participants.

| **Category** | **Code** | **Code Count**  (AGS/SGS/NP/SGI/AGI)* | **Sample Excerpt** |
| --- | --- | --- | --- |
| Perception vs. Reality | Sees more differences | 82  (30/10/31/6/5) | " It flowed better and highlighted important points." |
|  | Did not notice a difference | 21  (4/0/10/3/4) | "I didn't notice a difference until it was pointed out. I read everything thoroughly but I must've missed the gender change" |
| Discourse | Inclusiveness | 33  (3/2/2/6/21) | "Used non-gendered language ('people born with uterus and ovaries'), I liked this because it's more inclusive" |
|  | Bioessentialism | 26  (22/2/2/0/0) | "It BELITTLES WOMEN to use de-gendered language surrounding a biological process that literally only women can experience." |
|  | Trans/non-binary inclusiveness | 12  (0/0/1/4/7) | " As someone who is nonbinary and has PCOS, I prefer the version where gendered language has been removed as it speaks more to me as a person." |
|  | Media Tropes (bold type) | 11  (9/0/2/0/0) | " I don't agree with statements such as people with a uterus. It is man or woman. I am 100% woman and am proud of that. Stop **erasing** **women**."  "It uses female terms. It **admits** **truth** that those who would use the Pill are FEMALE." |
|  | Emotional Valence | 7  (6/0/1/0/0) | “[…] if it [*the healthcare system*] cannot step up and say yes, male and female and with some rare deviations albeit rare, we are doomed as humanity.” |
|  | Scientific Accuracy | 5  (4/0/1/0/0) | "It was scientifically accurate and didn't pretend like men or 'non-women' could have ovaries" |
| Inclusiveness Evaluation | Benefits of inclusive | 27  (3/2/0/4/17) | " Using 'people' instead of 'girls and women' is less distracting. Not only is it more inclusive, but I also think it's just a good editorial decision. Like, just say 'sperm' or 'ovaries.'" |
|  | Benefits of gender specific) | 14  (9/4/0/1/0) | " I preferred the gendered language because it made me feel more centered and considered as a woman." |
|  | Does not want inclusive | 12  (9/2/1/0/0) | " It referred to those who can get PCOS as women and girls, which is what they are. Not, 'people with ovaries.'" |
|  | Balanced pros and cons | 8  (3/2/1/2/0) | "The materials effectively highlighted the difference between gender-specific and de-gendered language. The de-gendered approach felt inclusive and respectful but occasionally required more effort to process. Balancing inclusivity with clarity is important to ensure accessibility for all readers." |
| Personal Preference | Wants gender specific | 14  (12/1/0/1/0) | " I am not opposed to gender neutral information and materials; however, I feel the omission of the words 'woman/girls' is harmful and misleading when educating people on health care issues affecting woman." |
|  | Wants inclusive | 13  (1/0/1/2/9) | "Using gender neutral language won't even register to most people, but it will make a difference to those who don't identify within the societal norms of gender identity" |
| Identity | LGBTQ+ | 13  (3/0/1/2/7) | "As a member of the LGBTQ+ community, I believe that transgender identity and issues deserve their own distinct recognition. […] Understanding and mutual respect are key, and I think the conversation around these topics benefits from nuance and openness rather than insistence on universal agreement." |
|  | Trans/non-binary | 7  (1/0/1/1/4) | "I liked that it said people who have ovaries, as I have ovaries but don't consider myself a woman" |
|  | Woman | 5  (3/0/1/1/0) | "[ …]I don't particularly hv[*sic*] an issue, it just makes me feel 'seen' as a natural born, 'Cis'gendered woman. I'm just a woman. Thassit lol[*sic*] " |
| Writing Quality | Clarity/wordiness | 6  (2/1/0/2/1) | "[…] However, the balance between inclusivity and clarity is crucial. While de-gendered language can make materials more accessible, it's essential to avoid ambiguity, especially when addressing specific biological or medical needs. […]" |

*****Code breakdown by number of excerpts from each explicit preference category (Always Gender-Specific/Sometimes Gender-Specific/No Preference/Sometimes Gender-inclusive/Always Gender-inclusive
